# MTR4: A Conserved Youth Factor for Vascular Endothelium

**DOI:** 10.1111/acel.70774

**Published:** 2026-10-16

**Authors:** Zhenyuan Guo, Zhiqiang Chen, Jiaqi Sun, Xiaojun Zhou, Chengzhi Ni, Yuxiang Zhang, Pengfei Qiang, Lu Zhang

**Affiliations:** ^1^ College of Bioengineering Henan University of Technology Zhengzhou China

## Abstract

Vascular endothelial cell (VEC) senescence drives organismal aging and age‐related pathologies, yet the intrinsic factors preserving endothelial youthfulness remain poorly defined. Here, we identify MTR4, an evolutionarily conserved RNA helicase and nuclear RNA exosome cofactor, as a novel regulator of VEC senescence. MTR4 protein declines markedly in senescent HUVECs and aged mouse aortas, independent of general cell cycle exit. Mechanistically, senescence‐induced upregulation of the E3 ubiquitin ligase synoviolin 1 (SYVN1) promotes K638‐linked ubiquitination and proteasomal degradation of MTR4. MTR4 depletion drives proliferating HUVECs into premature senescence, whereas its overexpression restores a youthful phenotype in senescent cells by binding BUB3 to stabilize BUB3 and sustain BUBR1 expression. In mice, endothelial Mtr4 knockdown promotes vascular dysfunction and multi‐organ aging, while endothelium‐specific Mtr4 overexpression alleviates these phenotypes. In *C. elegans* , *mtr‐4* overexpression dramatically extends lifespan, comparable to classic longevity models and surpassing most single‐gene interventions, while its knockdown accelerates aging. Our findings establish MTR4 as a critical guardian of endothelial homeostasis and organismal aging, with strong translational potential for mitigating age‐related vascular dysfunction.

## Graphical abstract

Senescence‐induced SYVN1 degrades MTR4 via K638 ubiquitination. MTR4 loss disrupts its binding to BUB3 and destabilizes BUB3, diminishing BUBR1 and driving endothelial senescence. MTR4 restoration reverses vascular aging in mice and extends C. elegans lifespan.

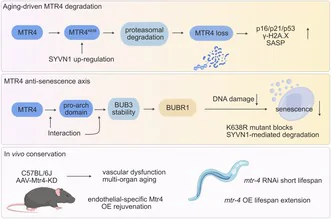

## 1 Introduction

Vascular endothelial cells (VECs) form the inner lining of blood vessels and act as a critical interface between the circulation and surrounding tissues. Beyond their role as a physical barrier, VECs dynamically regulate vascular tone, permeability, inflammation, and hemostasis, thereby maintaining multi‐organ homeostasis and modulating disease pathogenesis (Augustin and Koh 2024; Bloom et al. 2023; Ding et al. 2025). Chronically exposed to hemodynamic and metabolic stress, VECs are among the first cell types to acquire age‐related damage (Bloom et al. 2023). Senescent VECs undergo irreversible cell cycle arrest and acquire a pro‐inflammatory senescence‐associated secretory phenotype (SASP), which propagates local and systemic chronic inflammation. Endothelial dysfunction is now recognized as a central driver of major age‐related disorders including atherosclerosis, diabetes, and neurodegeneration, yet its contribution to age‐related mortality remains underappreciated (Han and Kim 2023).

Genetic evidence has established a causal role for endothelial aging in systemic physiological decline. Endothelium‐specific expression of progerin in mice recapitulates features of accelerated systemic aging and shortens lifespan similarly to ubiquitous progerin expression (Sun et al. 2020). Likewise, our previous work showed that endothelium‐targeted induction of cellular senescence is sufficient to reduce lifespan in mice (Zhang et al. 2023). Patients with Hutchinson‐Gilford progeria syndrome exhibit premature VEC senescence and lethal vascular complications (Laarmann et al. 2019; McClintock et al. 2006), whereas reversing endothelial aging extends lifespan in progeroid mice by approximately 20% (Mojiri et al. 2021). Together, these findings support the geroscience hypothesis that targeting fundamental aging processes may be more effective for preventing age‐related multimorbidity than treating individual diseases separately (Sierra and Kohanski 2017). Given their central role in systemic health, VECs represent an attractive target for antiaging interventions whose modulation could preserve homeostasis and reduce age‐related disease risk.

Current anti‐senescent strategies are broadly categorized as senolytics (which clear senescent cells) or senomorphics (which attenuate the SASP) (Gasek et al. 2021). Although effective in preclinical models, no such agents are clinically approved specifically for vascular endothelial senescence. Moreover, emerging evidence highlights important limitations of both approaches. Sustained elimination of senescent VECs can impair blood‐tissue barrier integrity, promote fibrosis (Grosse et al. 2020), and worsen pathology in pulmonary hypertension and liver injury models (Born et al. 2023; Zhao et al. 2024). Conversely, broad SASP suppression may interfere with immune surveillance and delay tissue repair (Chibaya et al. 2023; Demaria et al. 2014). These observations collectively suggest that bolstering the intrinsic resilience of VECs may represent a safer and more sustainable therapeutic strategy. Enhancing endogenous endothelial protective pathways improves metabolic health, alleviates vascular pathology, and extends healthspan (Chivite et al. 2026; Pang et al. 2025). Identifying novel endogenous factors that preserve vascular endothelial youthfulness thus represents a high‐priority goal. To date, most studies have focused on a limited set of factors such as Klotho and SIRT family proteins (Bloom et al. 2023; Das et al. 2018; Pang et al. 2025), leaving a critical gap in our understanding of the regulatory network governing VEC senescence.

MTR4 (SKIV2L2) is an evolutionarily conserved RNA helicase and core cofactor of the nuclear RNA exosome (Olsen and Johnson 2021). It plays a key role in RNA metabolism by associating with NEXT, PAXT, and TRAMP complexes, targeting nuclear RNAs for processing and degradation to maintain nuclear RNA homeostasis (Buzovetsky and Klinge 2025; Olsen and Johnson 2021; Schmid and Jensen 2019). Recent studies have identified additional functions of MTR4 beyond RNA metabolism. MTR4 facilitates rRNA processing to regulate the RPL5‐MDM2‐p53 signaling axis and control cell cycle progression (Xie et al. 2025). Depletion of MTR4 induces G2/M arrest in neuroblastoma cells accompanied by aberrant accumulation of replication‐dependent histone mRNAs (Onderak and Anderson 2017). It is also essential for meiotic initiation, as its loss blocks the mitotic‐to‐meiotic transition in spermatogonia (Zhang, Wang, et al. 2025). Furthermore, inactivation of EXOSC2, a core exosome subunit that acts in concert with MTR4, drives embryonic stem cells toward a pro‐senescent state characterized by nuclear RNA accumulation and transcriptional repression (Han et al. 2024). Together, these findings suggest a potential connection between MTR4 and cellular senescence. However, whether MTR4 directly regulates senescence, particularly in the aging vascular endothelium, remains completely unknown.

In the present study, we observed a marked reduction in MTR4 protein levels in senescent human umbilical vein endothelial cells and aortas from aged mice. We therefore explored the functional role of MTR4 in VEC senescence and investigated the underlying molecular mechanisms.

## 2 Materials and Methods

### 2.1 Materials

All antibodies, reagents, animals, plasmids, primers, and software are listed in Table S1.

### 2.2 Cell Culture and Treatments

HUVECs (Cell Bank of CAS) were cultured on gelatin‐coated dishes in ECM (5% FBS, 1× endothelial growth supplements, 100 U/mL penicillin—streptomycin) at 37°C, 5% CO_2_. HEK293T cells were maintained in high‐glucose DMEM (10% FBS, antibiotics) without gelatin. HUVECs were identified by cobblestone morphology and CD31 positivity. Replicative senescence was induced by serial passaging until growth arrest; stress‐induced premature senescence was induced by 200 μM H_2_O_2_ or 1 μM 5‐fluorouracil for 24 h.

### 2.3 Western Blot

Whole‐cell lysates (20 μg protein/well) were mixed with loading buffer (25 mM Tris–HCl pH 6.8, 2% SDS, 6% glycerol, 1% 2‐mercaptoethanol, 0.02% bromophenol blue, 2 mM PMSF, protease inhibitor cocktail), boiled 10 min at 95°C, separated by SDS‐PAGE, transferred to nitrocellulose membranes, blocked, and probed with primary antibodies followed by IRDye 800‐conjugated secondary antibodies. Signals were scanned/quantified with LI‐COR Odyssey. Antibody details in Table S1.

### 2.4 qRT‐PCR

Total RNA from HUVECs (NcmSpin Kit) or *C. elegans* (NGzol Kit) was reverse‐transcribed using HiScript III All‐in‐one RT SuperMix (Vazyme, R333). qPCR was performed with SYBR Green and specific primers (Table S1).

### 2.5 SA‐β‐Gal

Staining was performed using Solarbio G1580 kit per instructions. Adherent cells were processed directly; fresh mouse tissue blocks were fixed in kit fixative. Samples were incubated with freshly prepared staining working solution at 37°C (non‐CO_2_) overnight and observed under a Leica DM IL LED Fluo microscope.

### 2.6 Immunohistochemistry

Mouse aortic arches were fixed in 4% PFA, paraffin‐embedded, and sectioned (4‐μm). Sections were deparaffinized, rehydrated, permeabilized (0.3% Triton X‐100), blocked (2% BSA‐PBS, 30 min, RT), and incubated with anti‐Mtr4 (1:1000, overnight, 4°C) followed by HRP‐conjugated secondary (1:200, 1 h, RT). DAB development and hematoxylin counterstaining were performed. Images captured with Pannoramic 250 FLASH; DAB quantified by ImageJ 2.3.0.

### 2.7 Plasmids

CDS of *MTR4* and mutants were cloned into pCMV‐3xMyc; CDS of *MYC*, *BUB3*, *FEN1*, *CDK1*, *AIFM1* into pCMV‐3xFlag. shRNA targeting *MTR4* or *SYVN1* was inserted into PLKO.1. AAV‐based pAAV‐U6‐*Mtr4*‐shRNA1, pAAV‐U6‐*Mtr4*‐shRNA2, and pAAV2‐ICAM2‐*Mtr4* overexpression plasmids were purchased. Sequences in Table S1.

### 2.8 Co‐IP

Cells were lysed in RIPA buffer (50 mM Tris–HCl pH 7.4, 150 mM NaCl, 5 mM EDTA, 1% NP‐40, 0.5% sodium deoxycholate, 10 μg/mL aprotinin/leupeptin, 1 mM PMSF). For endogenous Co‐IP, HUVEC lysates were incubated with anti‐MTR4 or IgG overnight at 4°C, captured with Protein A/G agarose beads (1 h, 4°C), washed, and analyzed by Western blot. For overexpression Co‐IP, HEK293T cells co‐transfected with Myc‐MTR4 (or mutant) and Flag‐tagged candidates were lysed and incubated with anti‐Myc magnetic beads overnight; bound proteins were detected with anti‐Flag.

### 2.9 Lentivirus Preparation and Transduction

Lentivirus was packaged in HEK293T cells (80% confluence) using a three‐plasmid system (transfer vector, psPAX2, pMD2.G) and PEI transfection. DNA mixture (10 μg transfer + 7.5 μg psPAX2 + 5 μg pMD2.G) and PEI (1 μg/mL, pH 7.0) were each diluted in 500 μL Opti‐MEM, mixed, incubated 20 min at RT, and added to medium. Medium changed at 6–8 h. Viral supernatants were collected at 48 and 72 h, filtered (0.45 μm), concentrated by ultracentrifugation, and resuspended in PBS. HUVECs at 70%–80% confluence were transduced with concentrated virus and 8 μg/mL polybrene.

### 2.10 RNA‐Seq

Total RNA was extracted with TRIzol. Libraries and sequencing (Illumina NovaSeq) were performed by Wuhan Jingkairui. Raw reads were QC‐filtered with FASTQC, aligned to hg38/GRCh38 using HISAT2, and counted with FeatureCounts. DEGs (*p* < 0.05, |fold change| > 1.5) were identified by DESeq2 (v1.16.1); pathway enrichment via REACTOME.

### 2.11 MS

Myc‐MTR4 was overexpressed in HEK293T cells and immunoprecipitated with Myc magnetic beads (Biolinkedin, L‐1102A) in RIPA buffer. Enriched samples were analyzed by Qinglian Baiao using Q Exactive HF‐X; data searched against human proteome (UniProt SP UP000005640) with Proteome Discoverer 2.4. For candidate screening, proteins detected with more than 3 peptides in both the experimental and control groups were retained, and the remaining experimental‐unique proteins were then considered candidate interactors.

### 2.12 *C. elegans* Culture and RNAi

N2 wild‐type, *eat‐2*, *daf‐2*, and *mtr‐4* overexpressing transgenic worms were cultured at 20°C on NGM plates seeded with OP50. For RNAi, dsRNA‐expressing HT115 strains (target or empty L4440 control) were grown in LB (100 μg/mL ampicillin), concentrated, and plated on NGM with 1 mM IPTG and 100 μg/mL ampicillin (induced ≥ 2 h at 25°C) before worm transfer. RNAi reagents were replenished every 48 h. Targeting sequences in Table S1.

### 2.13 Lipofuscin and Lipid Accumulation

Synchronized eggs were cultured on OP50 at 20°C, and then transferred daily from the L1 stage to NGM plates seeded with HT115 bacteria (containing L4440 empty vector or RNAi) until adult day 7. Thirty worms were mounted on 2% agarose pads with 5 mM sodium azide for lipofuscin autofluorescence imaging using an Axio Observer 3 microscope. For Oil Red O staining, worms were washed in M9 buffer containing 0.05% Triton X‐100, fixed in 60% isopropanol for 3 min, centrifuged at 560 × g for 30 s, incubated with filtered Oil Red O solution overnight at 4°C, and finally imaged under a light microscope (Nikon ECLIPSE E200MV RS).

### 2.14 Pharyngeal Pumping and Body Bending

Synchronized eggs were cultured on OP50 at 20°C, and then transferred daily from the L1 stage to NGM plates seeded with HT115 bacteria (containing L4440 empty vector or RNAi) until adult day 7. Worms were observed under a stereomicroscope (Leica M205). For each group of approximately 30 worms, the pharyngeal pumping rate and body bends were counted within 30 s, and locomotion was recorded with a CCD camera (> 30 s per worm).

### 2.15 Lifespan Assay

Synchronized eggs were cultured on OP50 at 20°C, then transferred daily from L1 to HT115 plates (L4440 or RNAi). After egg‐laying cessation, worms were transferred every 2–3 days until death (scored by no response to platinum wire touch). Escapers, internal hatchlings, ruptured, or off‐plate worms were excluded. Each group started with ≥ 100 worms (*n* = 100–120). Survival recorded daily.

### 2.16 Stress Resistance and ROS

Synchronized eggs were cultured on OP50 at 20°C and then transferred daily from the L1 stage to NGM plates seeded with HT115 bacteria (containing L4440 empty vector or RNAi) until adult day 7. stress resistance was tested by exposure to 6 mM tBOOH or 35°C; death recorded every 2 h. For ROS, 80 worms per group were washed in M9, incubated with 50 μM DCFH‐DA (dark, 30 min), and fluorescence (ex/em 488/525 nm) was measured every 2 min for 30 min using a TECAN Spark microplate reader.

### 2.17 Mice and Adenovirus Administration

C57BL/6J mice (Zhengzhou University) were housed under SPF conditions. All procedures were approved by the Animal Ethics Committee (approval No. ZZU‐LAC20250219[01]). Adenovirus was packaged in HEK293T cells with three plasmids (target, pHelper, pRC9) using PEI. At 72 h, supernatant was collected, mixed with 6% PEG‐8000/0.8 M NaCl (overnight, 4°C), centrifuged (10,000 × g, 30 min, 4°C), and pellet resuspended in PBS. Viral titer was determined by qPCR; 2 × 10^12^ vg/kg was injected via tail vein.

### 2.18 H&E Staining

Tissue samples (heart, liver, spleen, lung, and kidney) were fixed in 4% PFA (24 h), dehydrated, cleared, and paraffin‐embedded. Sections (5 μm) were deparaffinized, rehydrated, stained with hematoxylin (8 min), differentiated in 1% acid ethanol, blued, counterstained with eosin (2 min), dehydrated, cleared, and mounted. Images were captured with a light microscope (SWE‐CX63, Servicebio).

### 2.19 Statistical Analysis

Data were analyzed using unpaired two‐tailed *t*‐test, one‐way or two‐way ANOVA with post hoc tests (GraphPad Prism 9). Survival curves were compared by log‐rank (Mantel‐Cox) test. Data are mean ± SD, significance at *p* < 0.05. Replicates and sample sizes are in figure legends. Western blot intensities were quantified with Image Studio Digits 5.2.5.

### 2.20 Data Availability

RNA‐seq data have been deposited in GEO database (GSE326321); MS interactome data have been deposited in ProteomeXchange database (PXD076350).

## 3 Results

### 3.1 MTR4 Expression Declines During VEC Senescence

To investigate the potential role of MTR4 in VEC senescence, we first examined its expression across multiple senescence models. Replicative senescence was induced in HUVECs from three independent donors via serial passaging; this was confirmed by increased senescence‐associated β‐galactosidase (SA‐β‐gal) activity and upregulated p16 and p21 levels (Figure 1A,B). In these senescent HUVECs, MTR4 protein levels were consistently and significantly reduced across all donors (Figure 1B). A similar decrease in MTR4 protein was observed in stress‐induced premature senescence models, including H_2_O_2_‐induced oxidative stress and 5‐fluorouracil (5‐FU)‐induced DNA damage (Figure 1C,D). To distinguish whether this MTR4 downregulation is specific to senescence rather than a general consequence of cell cycle exit, we measured MTR4 levels in quiescent, contact‐inhibited proliferating HUVECs and detected no significant changes (Figure 1E), confirming the specificity of MTR4 reduction to the senescent phenotype.

**FIGURE 1:**
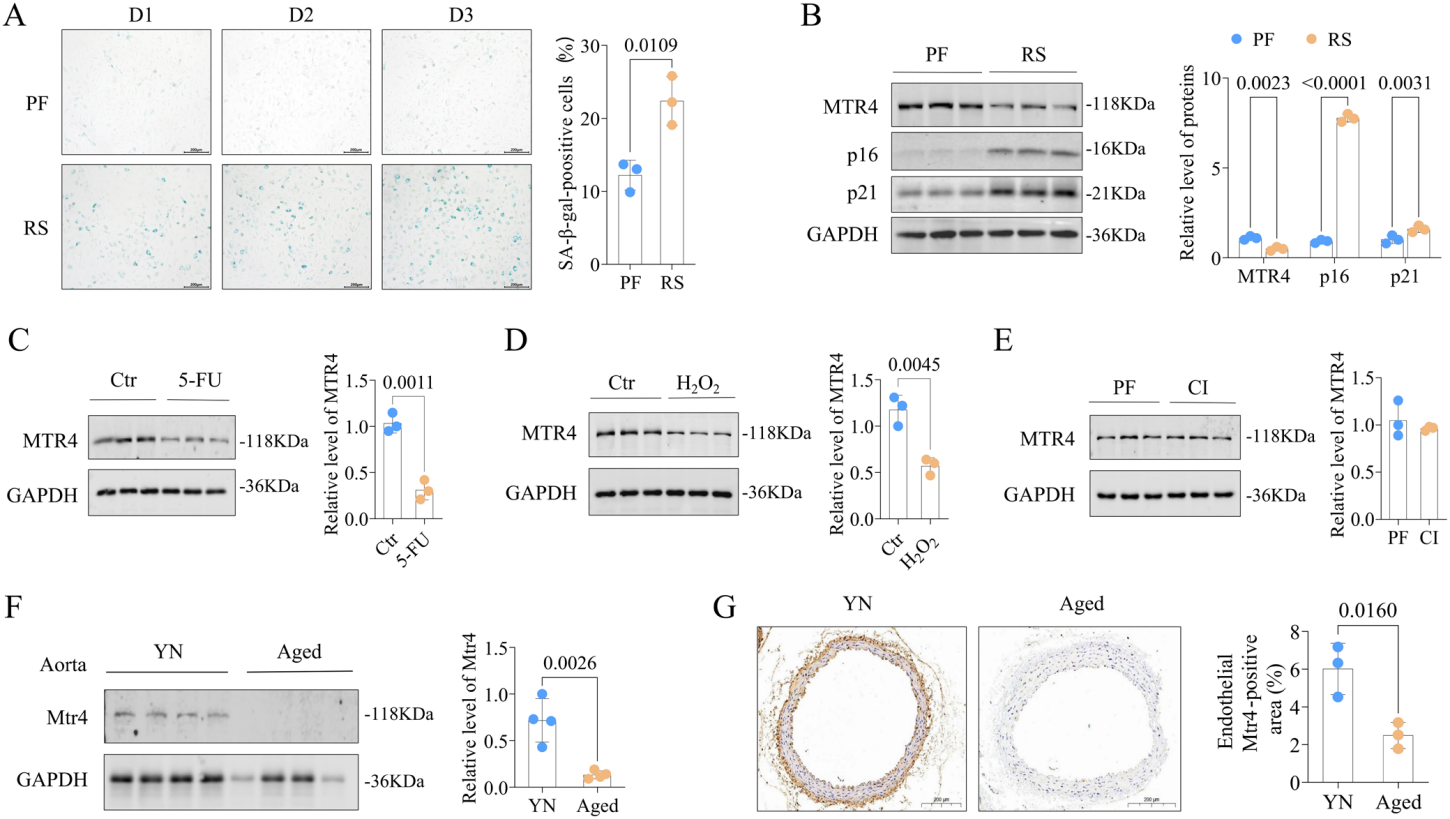
MTR4 expression declines during VEC senescence. (A) SA‐β‐gal staining of proliferating (PF) and replicative senescent (RS) HUVECs. D represents donor (scale bar, 200 μm) (*n* = 3). (B) Western blot analysis of MTR4, p16, and p21 protein levels in PF and RS HUVECs (*n* = 3). (C–E) Western blot analysis of MTR4 protein levels in HUVECs treated with 5‐FU or H_2_O_2_, or under contact inhibition (CI) (*n* = 3). (F) Western blot analysis of MTR4 protein levels in aortas of young (3–5 months) and aged (18–20 months) mice (*n* = 4). (G) Immunohistochemical staining of MTR4 in aortas of young and aged mice (scale bar, 200 μm) (*n* = 3). Data are presented as mean ± SD. *p* Values were determined by unpaired two‐tailed *t*‐test with Bonferroni correction for multiple comparisons.

We next validated these in vitro findings in vivo. Compared to young (3‐month‐old) mice, aged (20‐month‐old) mice exhibited a significant reduction in Mtr4 protein not only in the aorta but also in the heart, liver, spleen, and lung, with no significant change observed in the kidney (Figure 1F, Figure S1). Immunohistochemical staining further confirmed decreased Mtr4 immunoreactivity in the aortic endothelium of aged mice (Figure 1G). Furthermore, we re‐analyzed the Tabula Muris Senis single‐cell dataset (GSE149590) (Tabula Muris Consortium 2020), focusing on coronary artery endothelial cells. To stratify cells by senescence status, we employed the hUSI_score (Wang et al. 2025), a transcriptomic signature of cellular senescence, which we mapped to mouse one‐to‐one orthologs via Ensembl BioMart. Coronary endothelial cells were divided into non‐senescent (bottom 25%, *n* = 682) and senescent (top 25%, *n* = 659) populations based on hUSI_score. Notably, *Mtr4* mRNA expression was significantly lower in senescent endothelial cells than in their non‐senescent counterparts (Figure S2). This single‐cell analysis demonstrates that MTR4 downregulation is a cell‐autonomous feature of endothelial senescence in vivo, independent of alterations in tissue cellular composition. Collectively, these data demonstrate that MTR4 downregulation is a consistent feature of VEC senescence both in vitro and in vivo.

### 3.2 MTR4 Regulates Senescence in Cultured HUVECs

To determine whether reduced MTR4 contributes to VEC senescence, we manipulated MTR4 expression in proliferating HUVECs. Lentiviral shRNA‐mediated knockdown of MTR4 significantly increased the proportion of SA‐β‐gal‐positive cells, upregulated the levels of senescence markers (p16, p21, p53, and γ‐H2A.X), and decreased endothelial nitric oxide synthase (eNOS) levels (Figure 2A,B). Additionally, MTR4 knockdown upregulated the mRNA levels of multiple SASP factors, including the proinflammatory cytokines *IL‐1α* and *IL‐6*, the chemokine *CCL2*, the growth factor *TGF‐β*, the matrix‐remodeling enzyme *MMP3*, and *TNF‐α* (Figure 2C).

**FIGURE 2:**
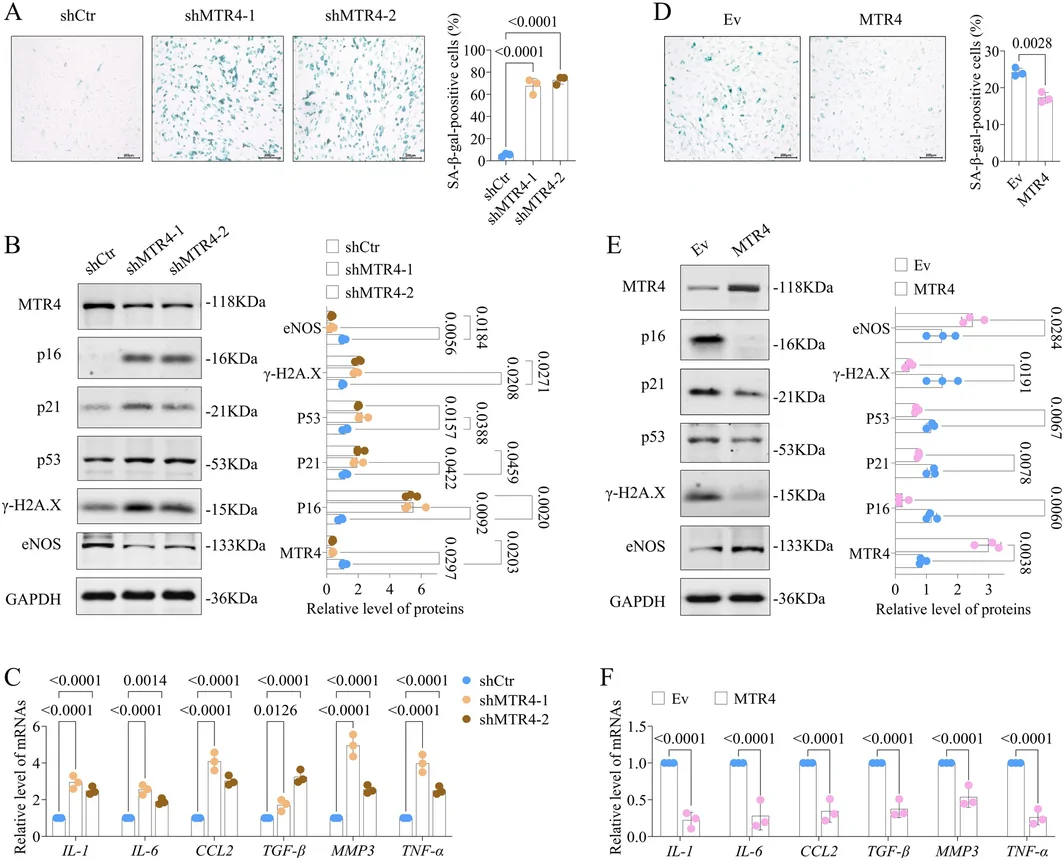
MTR4 regulates senescence in HUVECs. (A) SA‐β‐gal staining of HUVECs after MTR4 knockdown (scale bar, 200 μm) (*n* = 3). (B) Western blot analysis of the levels of senescence markers (p16, p21, p53, γ‐H2A.X, and eNOS) in HUVECs after MTR4 knockdown (*n* = 3). (C) RT‐qPCR analysis of the levels of SASP factors (*IL‐1*, *IL‐6*, *CCL‐2*, *TGF‐β*, *MMP3*, and *TNFα*) in HUVECs after MTR4 knockdown (*n* = 3). (D) SA‐β‐gal staining of HUVECs after MTR4 overexpression (scale bar, 200 μm) (*n* = 3). (E) Western blot analysis of the levels of senescence markers (p16, p21, p53, γ‐H2A.X, and eNOS) in HUVECs after MTR4 overexpression (*n* = 3). (F) RT‐qPCR analysis of SASP factor levels in HUVECs after MTR4 overexpression (*n* = 3). Data are presented as mean ± SD. *p* Values were determined by one‐way ANOVA followed by Dunnett's multiple comparisons test (A–C). by unpaired two‐tailed *t*‐test with Bonferroni correction for multiple comparisons (D–F).

Conversely, MTR4 overexpression in serially passaged, senescent HUVECs attenuated the senescent phenotype. This was evidenced by a reduced number of SA‐β‐gal‐positive cells, decreased levels of senescence markers, preserved eNOS levels, and suppressed upregulation of SASP factors (Figure 2D–F). Collectively, these results demonstrate that MTR4 is not only correlated with VEC senescence but also functionally regulates this process.

### 3.3 MTR4 Binds and Stabilizes BUB3 to Suppress VEC Senescence

To explore the molecular mechanism by which MTR4 regulates VEC senescence, we performed RNA‐seq analysis on control and *MTR4*‐knockdown HUVECs. MTR4 knockdown resulted in 938 upregulated and 1318 downregulated genes (GEO accession: GSE3263210, Figure S3A). Gene enrichment analysis revealed that upregulated genes were significantly enriched in Rho GTPase, GPCR, receptor tyrosine kinase, and MAPK signaling pathways, as well as SASP‐related processes including extracellular matrix organization (Figure S3B). In contrast, downregulated genes were primarily associated with cell cycle progression, DNA replication, and telomere maintenance (Figure S3C). The downregulation of genes within the “cellular senescence” pathway was largely attributed to broad suppression of core cell cycle regulators (e.g., CCNA2 and CDK2), chromatin assembly‐related genes (e.g., histone family members), and APC/C complex subunits, changes consistent with cell cycle exit and the senescent phenotype.

While transcriptomic analysis identified downregulation of cell cycle pathways following MTR4 knockdown, these changes likely represent indirect downstream effects. To identify direct protein interactors of MTR4, we performed immunoprecipitation coupled with mass spectrometry (IP‐MS), which identified 92 potential interacting proteins (ProteomeXchange accession: PXD076350). Pathway enrichment analysis of these interactors showed significant enrichment in cell cycle‐related pathways, consistent with our transcriptomic findings (Figure 3A). Intersection of MTR4‐interacting proteins with the GenAge database (a curated database of aging‐related genes) identified five overlapping genes: MYC, BUB3, FEN1, CDK1, and AIFM1 (Figure 3B). Co‐immunoprecipitation (Co‐IP) assays confirmed physical interaction between MTR4 and BUB3, as well as MTR4 and FEN1, but not between MTR4 and the other three candidates (Figure 3C–G). Analysis of our RNA‐seq data revealed that MTR4, FEN1, and BUB3 mRNA levels were all downregulated following MTR4 knockdown (Figure 3H). We validated these findings in *MTR4*‐knockdown HUVECs, confirming decreased FEN1 and BUB3 expression at both the mRNA and protein levels (Figure 3I,J). Furthermore, both FEN1 and BUB3 protein levels were reduced in replicatively senescent HUVECs (Figure 3K,L). To determine whether FEN1 or BUB3 mediates MTR4's effect on VEC senescence, we overexpressed each protein individually in *MTR4*‐knockdown HUVECs. Overexpression of *BUB3*, but not *FEN1*, significantly reduced the protein levels of senescence markers (Figure 3M,N). These data demonstrate that BUB3 is a key downstream effector of MTR4 in the regulation of VEC senescence.

**FIGURE 3:**
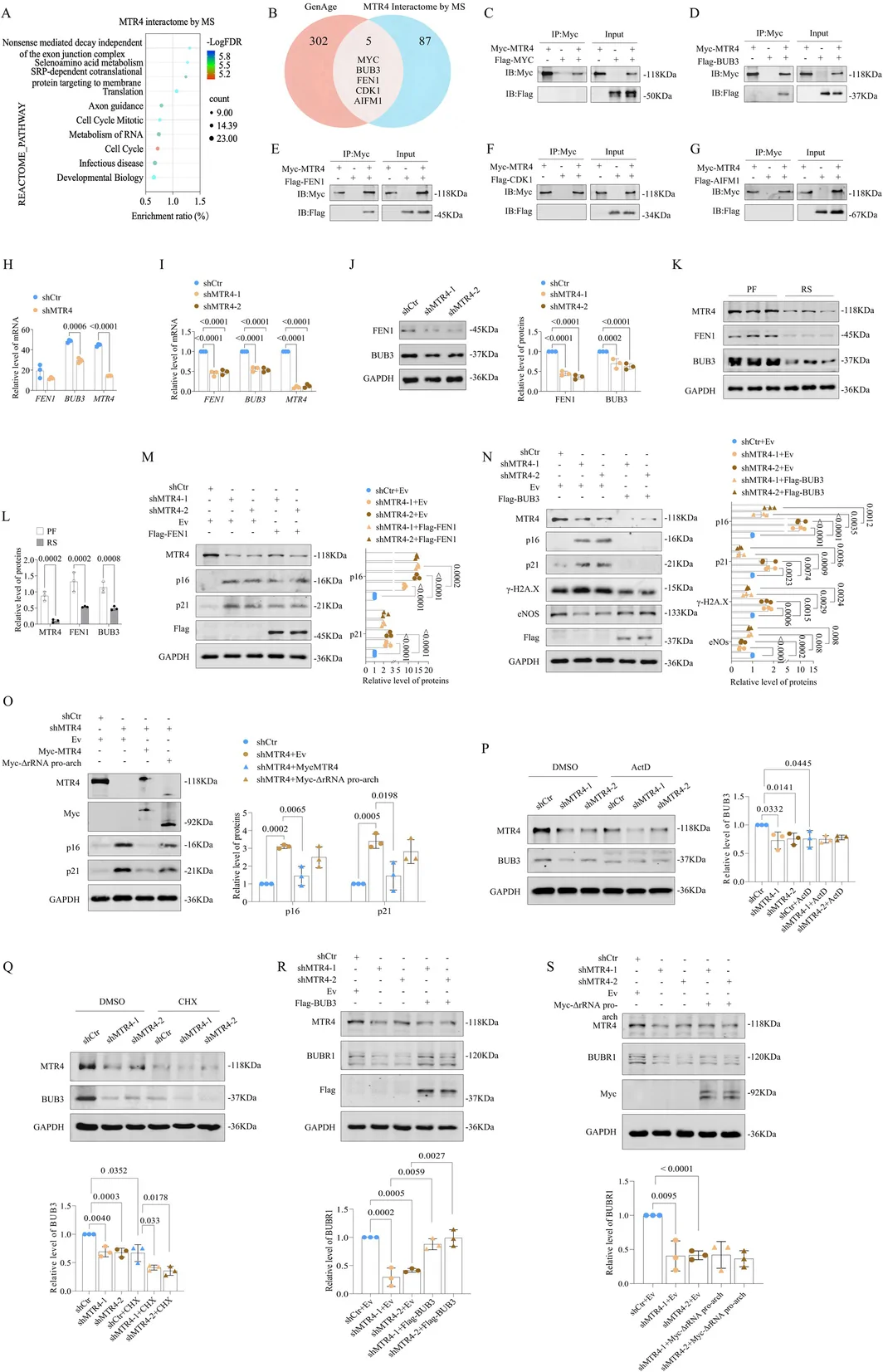
MTR4 binds and stabilizes BUB3 to suppress VEC senescence. (A) Bubble plot showing Reactome pathway enrichment analysis of MTR4‐interacting proteins identified via mass spectrometry in HEK293T cells overexpressing Myc‐MTR4. (B) Venn diagram illustrating the overlap between MTR4‐interacting proteins and genes in the GenAge database of aging‐related genes, highlighting five common candidates: MYC, BUB3, FEN1, CDK1, and AIFM1. (C–G) Co‐immunoprecipitation validation of the interaction between Myc‐MTR4 and Flag‐MYC, Flag‐BUB3, Flag‐FEN1, Flag‐CDK1, or Flag‐AIFM1 in HEK293T cells. (H) RNA‐seq analysis demonstrating reduced levels of *FEN1* and *BUB3* following MTR4 knockdown. (I) RT‐qPCR analysis of *FEN1* and *BUB3* mRNA levels in HUVECs after MTR4 knockdown (*n* = 3). (J) Western blot analysis of FEN1 and BUB3 protein levels in HUVECs after MTR4 knockdown (*n* = 3). (K, L) Western blot analysis of MTR4, FEN1, and BUB3 protein levels in proliferating versus replicatively senescent HUVECs (*n* = 3). (M) Western blot analysis of p16 and p21 protein levels in *MTR4*‐knockdown HUVECs following FEN1 overexpression (*n* = 3). (N) Western blot analysis of p16, p21, γ‐H2A.X, and eNOS protein levels in *MTR4*‐knockdown HUVECs following BUB3 overexpression (*n* = 3). (O) Western blot analysis of p16, p21 protein levels in *MTR4*‐knockdown HUVECs following Myc‐MTR4 or Myc‐ΔrRNA pro‐arch (*n* = 3). (P, Q) Western blot analysis of BUB3 protein levels in *MTR4*‐knockdown HUVECs treated with DMSO, ActD (5 μM) for 6 h, or CHX (10 μM) for 6 h (*n* = 3). (R, S) Western blot analysis of BUBR1 protein levels in *MTR4*‐knockdown HUVECs following Flag‐BUB3 or Myc‐ΔrRNA pro‐arch (*n* = 3). Data are presented as mean ± SD. *p* Values were determined by one‐way ANOVA followed by Dunnett's multiple comparisons test (J, M, N, P, Q, R, S) by unpaired two‐tailed *t*‐test with Bonferroni correction for multiple comparisons (H, K, O).

To determine the functional significance of the MTR4‐BUB3 interaction, we generated a truncation mutant of MTR4 lacking the rRNA pro‐arch domain, a region known to mediate protein–protein interactions without affecting MTR4's ATPase and helicase activities (Jackson et al. 2010; Lingaraju et al. 2019). Co‐immunoprecipitation confirmed that while wild‐type MTR4 efficiently bound BUB3, the ΔrRNA pro‐arch mutant completely lost this interaction (Figure S4). When we reconstituted *MTR4*‐knockdown HUVECs with either wild‐type MTR4 or the binding‐deficient mutant, only wild‐type MTR4, but not the ΔrRNA pro‐arch mutant, was able to rescue the senescence‐associated upregulation of p16 and p21 (Figure 3O). These results demonstrate that the physical interaction between MTR4 and BUB3 is functionally indispensable for MTR4's anti‐senescence activity.

We next examined how MTR4 regulates BUB3 protein levels. Upon Actinomycin D (ActD) treatment to block transcription, BUB3 protein levels were comparable between control and *MTR4*‐knockdown cells (Figure 3P), suggesting that MTR4 does not maintain BUB3 primarily through stabilizing its mRNA. In contrast, upon cycloheximide (CHX) treatment to block translation, BUB3 protein levels were significantly lower in *MTR4*‐knockdown cells than in control cells (Figure 3Q), indicating that MTR4 stabilizes BUB3 at the protein level.

To determine whether MTR4‐mediated BUB3 stabilization affects the BUB3‐dependent pathway, we examined BUBR1, a core spindle assembly checkpoint kinase whose stability strictly depends on BUB3 (Elowe et al. 2010; Overlack et al. 2015; Taylor et al. 1998). In *MTR4*‐knockdown HUVECs, BUBR1 levels were reduced, and this reduction was rescued by BUB3 (Figure 3R), but not by the binding‐deficient ΔrRNA pro‐arch mutant (Figure 3S). Together with our observation that BUB3 overexpression rescues MTR4 silencing‐induced γ‐H2A.X upregulation (Figure 3N), these data suggest that MTR4, through stabilizing BUB3, contributes to maintaining BUBR1 levels and limiting DNA damage, which may underlie its role in suppressing VEC senescence.

### 3.4 SYVN1 Mediates Senescence‐Associated Downregulation of MTR4 via K638 Ubiquitination

We next investigated the molecular basis underlying reduced MTR4 protein levels during endothelial senescence. *MTR4* mRNA levels were increased in senescent HUVECs and unchanged in aortas from aged mice (Figure S5A,B), excluding transcriptional repression and implicating post‐transcriptional regulation. Furthermore, analysis of transcriptomic data from the Genotype‐Tissue Expression (GTEx) database in healthy human tissues revealed that *MTR4* mRNA levels showed no significant differences across aorta, coronary artery, tibial artery, and total vascular tissues (Figure S6), which further supports our findings in aged mouse aortas. To define the degradative pathway involved, HUVECs and mouse endothelial C166 cells were treated with cycloheximide (CHX) to block new protein synthesis, in combination with inhibitors targeting major protein degradation pathways. The proteasome inhibitor MG132 significantly stabilized MTR4 protein against CHX‐induced decay, whereas autophagy inhibitors (wortmannin, bafilomycin A1, and CA‐5f) had no effect (Figure 4A). These results indicate that MTR4 is constitutively degraded primarily through the ubiquitin‐proteasome system. Consistent with this, endogenous MTR4 immunoprecipitated from replicatively senescent HUVECs exhibited markedly higher ubiquitination levels compared with proliferating control cells (Figure 4B).

**FIGURE 4:**
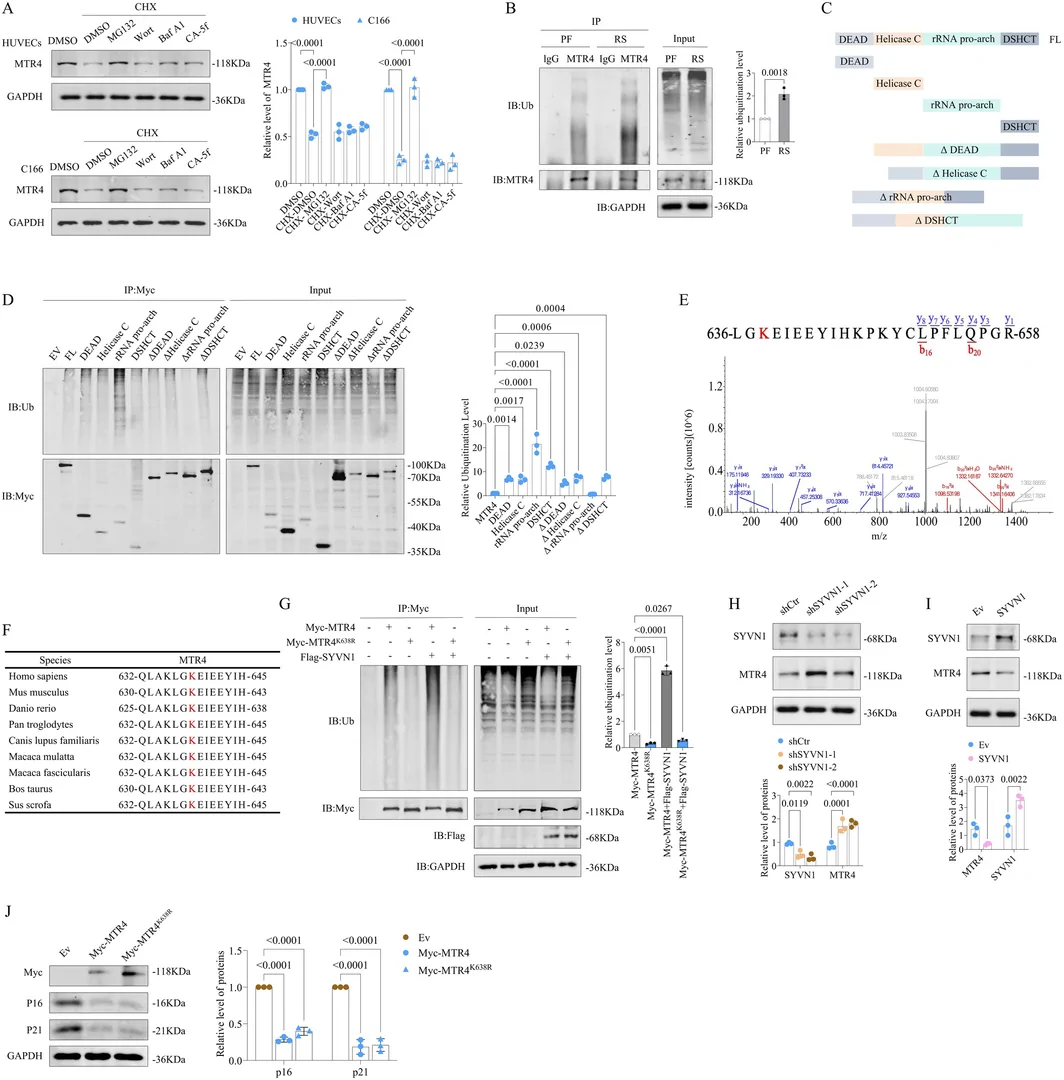
SYVN1 mediates senescence‐associated downregulation of MTR4 via K638 ubiquitination. (A) HUVECs and C166 cells were treated with CHX for 24 h, followed by treatment with MG132 (10 μM), the autophagy inhibitors wortmannin (100 nM), bafilomycin A1 (100 nM), or CA‐5f (20 μM) for 6 h. MTR4 protein levels were detected by Western blot (*n* = 3). (B) Co‐IP analysis of MTR4 ubiquitination levels in proliferating and replicative senescent HUVECs. (C) Schematic representation of MTR4 truncation mutants (DEAD, Helicase C, rRNA pro‐arch, DSHCT) and deletion mutants (ΔDEAD, ΔHelicase C, ΔrRNA pro‐arch, ΔDSHCT). (D) Co‐IP analysis of ubiquitination levels of MTR4 and its mutants (*n* = 3). (E) MS analysis of post‐translational modifications of Myc‐MTR4 in HEK293T cells overexpressing Myc‐MTR4, showing the MS/MS spectrum of residue K638. (F) Conservation of the MTR4 K638 residue across species. (G) Co‐IP analysis of ubiquitination levels in HEK293T cells overexpressing Myc‐MTR4, Myc‐MTR4K638R, Myc‐MTR4 together with Flag‐SYVN1, or Myc‐MTR4K638R together with Flag‐SYVN1 (*n* = 3). (H, I) Western blot analysis of MTR4 protein levels in HUVECs after SYVN1 knockdown or overexpression (*n* = 3). (J) Western blot analysis of P16, P21 protein levels in HUVECs after Myc‐MTR4 or Myc‐MTR4^K638R^ overexpression (*n* = 3). Data are presented as mean ± SD. *p* Values were determined by one‐way ANOVA followed by Dunnett's multiple comparisons test (A, D, G, H). by unpaired two‐tailed *t*‐test with Bonferroni correction for multiple comparisons (B, I, J).

To map the functional ubiquitination site on MTR4, we constructed a series of truncation and domain‐deletion mutants covering the four major structural domains of MTR4 (Figure 4C). Following transient transfection in HEK293T cells, a truncation mutant containing only the rRNA pro‐arch domain retained robust ubiquitination, while deletion of this domain nearly abolished MTR4 ubiquitination (Figure 4D). These data identify the rRNA pro‐arch domain as the principal region responsible for MTR4 ubiquitination. Subsequent mass spectrometry analysis of immunopurified MTR4 further pinpointed lysine 638 (K638) within this domain as a specific ubiquitination site (Figure 4E). Protein sequence alignment of MTR4 orthologs across diverse vertebrate species revealed that the surrounding motif (QLAKLGKEIEEYIH) harboring K638 is completely conserved from humans to pigs (Figure 4F), underscoring the functional importance of this residue in MTR4 regulation.

Previous reports have shown that SYVN1 acts as an E3 ubiquitin ligase for MTR4. We therefore tested whether SYVN1 mediates ubiquitination specifically at K638. Co‐expression of SYVN1 with wild‐type MTR4 in HEK293T cells strongly enhanced ubiquitination, an effect lost with the K638R mutant (Figure 4G), confirming specificity for this residue. SYVN1 protein levels were elevated in replicative senescent HUVECs (Figure S7), and knockdown of SYVN1 in these cells restored MTR4 (Figure 4H), and endogenous MTR4 (Figure 4I). Together, these results demonstrate that SYVN1 is upregulated during senescence and promotes MTR4 degradation via K638‐linked ubiquitination.

To directly test whether preventing MTR4 degradation is sufficient to reverse endothelial senescence, we utilized the ubiquitination‐deficient MTR4‐K638R mutant. Unlike wild‐type MTR4, which remains subject to SYVN1‐mediated degradation in senescent HUVECs, the K638R mutant evades ubiquitination at this critical residue. When overexpressed in replicatively senescent HUVECs, both wild‐type MTR4 and the MTR4‐K638R mutant significantly reduced p16 and p21 protein levels compared to vector control (Figure 4J). These findings demonstrate that specifically blocking K638‐linked ubiquitination and subsequent degradation of MTR4 is sufficient to preserve its anti‐senescence function, confirming that SYVN1‐mediated MTR4 downregulation is a causal driver of endothelial senescence.

### 3.5 Mtr4 Regulates Vascular and Multi‐Organ Aging in Mice

To investigate MTR4's role in mammalian aging, we modulated Mtr4 expression in mice (Figure S8). Both AAV‐mediated knockdown and overexpression of Mtr4 had no significant effect on body weight in mice (Figure S9). The open field test was performed to assess spontaneous locomotor activity and exploratory behavior, which are established indicators of age‐related behavioral decline in rodents (Elias et al. 1975; Shoji et al. 2016). Mtr4 knockdown in young mice impaired exploratory behavior, with reduced total distance traveled, fewer center zone entries, and lower average locomotor speed (Figure 5A–E). Endothelium‐specific Mtr4 overexpression in aged mice increased these exploratory behavior‐related parameters (Figure 5F–J). Vascular function assessment showed Mtr4 knockdown in young mice induced vascular dysfunction, with increased aortic arch blood flow velocity and reduced abdominal aortic systolic and diastolic diameters (Figure S10A,B). Endothelium‐specific Mtr4 overexpression in aged mice reversed these age‐associated vascular alterations (Figure S10C,D).

**FIGURE 5:**
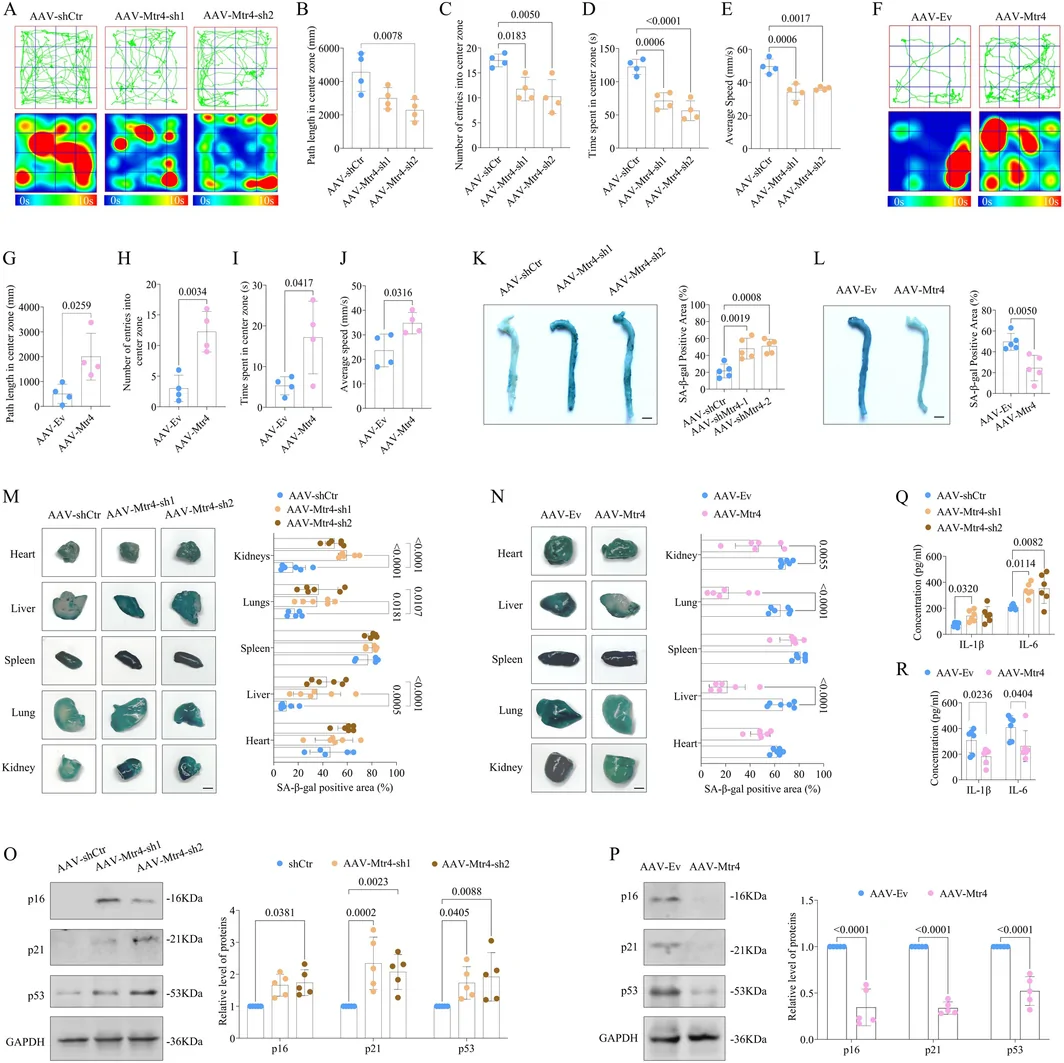
Mtr4 regulates vascular and multi‐organ aging in mice. (A–E) Open field test analysis of center zone path length, number of entries, time spent in the center zone, and average speed in young mice following AAV‐mediated Mtr4 knockdown (*n* = 4). (F–J) Open field test analysis of center zone path length, number of entries, time spent in the center zone, and average speed in aged mice following AAV‐mediated Mtr4 overexpression (*n* = 4). (K, L) SA‐β‐gal staining in aortas of young mice following Mtr4 knockdown and aged mice following Mtr4 overexpression (scale bar, 3 mm) (*n* = 5). (M, N) SA‐β‐gal staining in heart, liver, spleen, lung, and kidney tissues of young mice following Mtr4 knockdown and aged mice following Mtr4 overexpression (scale bar, 3 mm) (*n* = 5–6). (O, P) Western blot analysis of p16, p21, and p53 expression in mouse aortas from young mice following Mtr4 knockdown and aged mice following Mtr4 overexpression (*n* = 5). (Q, R) ELISA analysis of serum IL‐1 and IL‐6 levels in young mice following Mtr4 knockdown and aged mice following Mtr4 overexpression (*n* = 5–6). Data are presented as mean ± SD. *p* Values were determined by one‐way ANOVA followed by Dunnett's multiple comparisons test (B–E) (K, M, Q, O).by unpaired two‐tailed t‐test with Bonferroni correction for multiple comparisons (G–J) (L, N, R, P).

Histological analysis showed Mtr4 knockdown in young mice recapitulated age‐related morphological changes across organs. Spleen tissue exhibited irregular corpuscles, expanded marginal zones, and increased hemosiderin deposition, consistent with aged mice (Figure S11A). Liver tissue displayed hepatocellular vacuolization, a hepatic aging hallmark (Figure S11A). Lung tissue showed enlarged alveolar spaces and thickened septa, while kidneys had global glomerulosclerosis, typical of organ aging (Figure S11A). Aged mice with endothelium‐specific Mtr4 overexpression showed attenuation of these age‐related morphological changes, with tissue morphology similar to young controls (Figure S11B). SA‐β‐gal staining showed increased senescent cell accumulation, a key driver of organ aging, in the aorta, liver, lung, and kidney of *Mtr4*‐knockdown mice (Figure 5K,M). In contrast, endothelium‐specific Mtr4 overexpression in aged mice significantly reduced senescent cell accumulation in the aforementioned tissues to levels comparable to those of young controls (Figure 5L,N). Western blot analysis revealed that Mtr4 knockdown in young mice significantly increased the levels of the senescence marker proteins p16, p21, and p53, whereas endothelium‐specific Mtr4 overexpression in aged mice significantly decreased the levels of these senescence markers (Figure 5O,P). Serum ELISA revealed elevated IL‐1β and IL‐6 in *Mtr4*‐knockdown mice, and endothelium‐specific Mtr4 overexpression restored these cytokine levels to those of young controls (Figure 5Q,R). In addition, we examined BUB3 protein levels in the aortas of *Mtr4*‐knockdown mice. Consistent with our in vitro findings, BUB3 level was significantly reduced in the aortas of *Mtr4*‐knockdown mice compared with controls (Figure S12). Together, these data demonstrate that MTR4 maintains youthful homeostasis in mice; its depletion induces systemic senescence, while endothelium‐specific restoration of MTR4 counteracts age‐related phenotypes.

### 3.6 *mtr‐4* Regulates Aging and Lifespan in *Caenorhabditis elegans* ( *C. elegans* )

To determine whether MTR4's function in aging is conserved across species, we examined its homolog *mtr‐4* in *C. elegans* . The level of *mtr‐4* mRNA in aged worms was significantly decreased compared with young controls (Figure S5C). RNAi‐mediated knockdown of *mtr‐4* significantly shortened the lifespan of wild‐type worms (Figure 6A). The *mtr‐4* knockdown animals displayed accelerated age‐related functional decline, as indicated by reduced pharyngeal pumping rate and body bending frequency on day 7 of adulthood, along with diminished spontaneous movement (Figure 6B,C, Movies S1 and S2). Additionally, *mtr‐4* knockdown increased lipofuscin and lipid accumulation (Figure 6D,E), reduced survival under thermal and oxidative stress (Figure 6F,G), and elevated basal ROS levels (Figure 6H).

**FIGURE 6:**
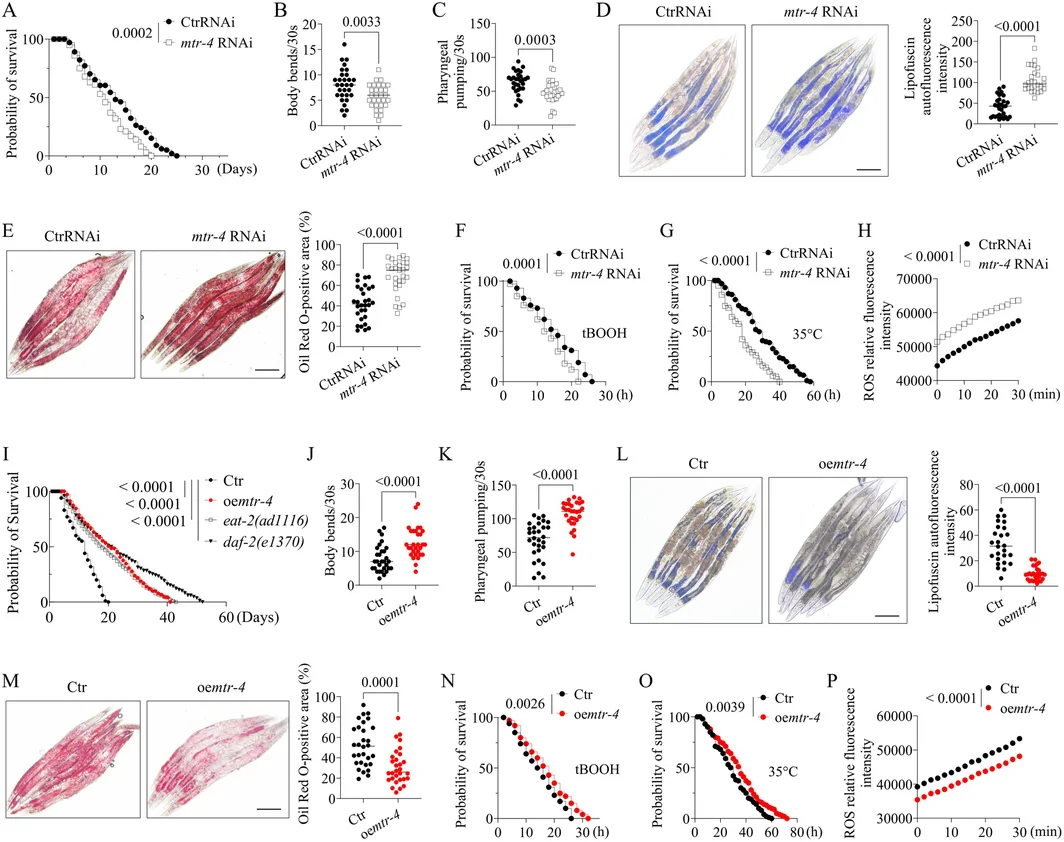
*mtr‐4* regulates aging and lifespan in *Caenorhabditis elegans* ( *C. elegans* ). (A) Lifespan analysis of N2 worms following *mtr‐4* knockdown (*n* = 100). (B, C) Body bend frequency and pharyngeal pumping frequency of N2 worms following *mtr‐4* knockdown (*n* = 30). (D, E) Lipofuscin autofluorescence intensity and Oil Red O staining area in N2 worms following *mtr‐4* knockdown (scale bar, 200 μm) (*n* = 30). (F–H) Thermotolerance (35°C), oxidative stress resistance (6 mM tBOOH), and ROS levels in N2 worms following *mtr‐4* knockdown. (I) Lifespan analysis of N2 worms following *mtr‐4* overexpression (*n* = 100). (J, K) Body bend frequency and pharyngeal pumping frequency of N2 worms following *mtr‐4* overexpression (*n* = 30). (L, M) Lipofuscin autofluorescence intensity and Oil Red O staining area in N2 worms following *mtr‐4* overexpression (scale bar, 200 μm) (*n* = 30). (N–P) Thermotolerance (35°C), oxidative stress resistance (6 mM tBOOH), and ROS levels in N2 worms following *mtr‐4* overexpression. Data are presented as mean ± SD. *p* Values were determined by unpaired two‐tailed t‐test with Bonferroni correction for multiple comparisons (B–E, J–M). by two‐way ANOVA followed by Tukey's multiple comparisons test (H, P). Lifespan curves were compared using the log‐rank (Mantel‐Cox) test.

Conversely, transgenic overexpression of *mtr‐4* in *C. elegans* extended lifespan to a degree comparable to that of the well‐characterized long‐lived *eat‐2(ad1116)* mutant (a genetic model of dietary restriction; Figure 6I). Worms overexpressing *mtr‐4* maintained higher locomotor activity in adulthood, as evidenced by preserved pharyngeal pumping and body bending frequencies (Figure 6J,K; Movies S3 and S4). Moreover, *mtr‐4* overexpression reduced age‐related lipofuscin and lipid accumulation (Figure 6L,M), improved survival under thermal and oxidative stress (Figures 5O and 6N), and lowered basal ROS levels (Figure 6P). Collectively, these data demonstrate that mtr‐4 is a conserved regulator of lifespan and age‐related functional decline, stress resistance, and cellular aging phenotypes in *C. elegans* .

## 4 Discussion

VEC senescence is a key driver of organismal aging and age‐related diseases, yet its underlying regulatory network remains incompletely understood. Here, we identify MTR4 as a conserved anti‐aging factor in the vascular endothelium. We show that MTR4 is downregulated during senescence via SYVN1‐dependent ubiquitination and proteasomal degradation, and that MTR4 counteracts endothelial senescence through physical interaction with BUB3. We further validate the conserved role of *mtr‐4/*Mtr4 in aging regulation in *C. elegans* and mice, supporting its potential as a therapeutic target for age‐related vascular and multi‐organ dysfunction.

Our findings establish MTR4 as a novel regulator of VEC senescence. Previous studies have focused on a limited set of endogenous antiaging factors in VECs, including Klotho and SIRT family proteins (Bloom et al. 2023; Das et al. 2018; Pang et al. 2025), whereas the functions of RNA helicases, which are critical guardians of RNA homeostasis, in endothelial senescence remain largely unexplored. MTR4 is a well‐characterized core cofactor of the nuclear RNA exosome that mediates nuclear RNA processing and decay (Buzovetsky and Klinge 2025; Olsen and Johnson 2021; Schmid and Jensen 2019), but its non‐canonical functions beyond RNA metabolism have only recently begun to emerge. Our data extend this understanding by demonstrating that MTR4 is significantly downregulated in senescent VECs both in vitro and in vivo, in a senescence‐specific manner independent of general cell cycle exit. Importantly, our single‐cell transcriptomic analysis of the Tabula Muris Senis atlas provides orthogonal validation at single‐cell resolution. Within the same aortic endothelial population from aged mice, senescent endothelial cells express significantly less *Mtr4* mRNA than their non‐senescent counterparts, confirming that MTR4 downregulation is a cell‐autonomous feature of senescence rather than a consequence of altered tissue composition. MTR4 is a functional determinant of cellular senescence. Its knockdown induces premature senescence in proliferating HUVECs, while its overexpression reverses the senescent phenotype in senescent HUVECs.

Using IP‐MS and functional rescue assays, we identified BUB3 as a key downstream effector mediating the antiaging effect of MTR4 and further defined how MTR4 regulates BUB3. Using ActD and CHX chase experiments, we show that MTR4 stabilizes BUB3 at the protein level rather than at the mRNA level. We mapped the MTR4‐BUB3 binding interface to the rRNA pro‐arch domain of MTR4, a structural feature distinct from its helicase core that serves as a protein–protein interaction platform (Jackson et al. 2010). Deletion of this domain completely abolishes BUB3 binding while preserving MTR4 catalytic activity (Lingaraju et al. 2019), and the ΔrRNA pro‐arch mutant fails to rescue senescence in *MTR4*‐knockdown cells. This directly demonstrates that the Arch‐dependent MTR4‐BUB3 interaction is indispensable for MTR4's anti‐senescence function. Downstream of BUB3, we further found that MTR4 maintains the spindle assembly checkpoint kinase BUBR1, whose stability strictly depends on BUB3 binding (Elowe et al. 2010; Overlack et al. 2015; Taylor et al. 1998). This finding links MTR4, an RNA helicase, to chromosome segregation and genome stability, providing a mechanistic explanation for how MTR4 deficiency triggers cellular senescence. We further observed that Mtr4 knockdown reduces BUB3 protein in mouse aortas, suggesting that this regulation also operates in vivo.

BUB3 is a conserved component of the spindle assembly checkpoint essential for accurate chromosome segregation (Silva and Bousbaa 2022). Emerging evidence supports additional roles in aging that are consistent with our observations. BUB3 has been shown to regulate vascular cell growth and survival via non‐canonical HIPPO/MST signaling and FOXO (Kudryashova et al. 2022), providing a potential mechanism for its involvement in vascular aging. The BUB3‐BUB1 complex facilitates telomere DNA replication (Li et al. 2018), a core process counteracting cellular senescence, suggesting a possible route through which the MTR4‐BUB3 axis maintains endothelial function. BUB3 also participates in RNA splicing, and its deficiency induces R‐loop accumulation, DNA damage, and p53‐dependent premature senescence (Wan et al. 2015). This agrees with our transcriptomic data showing that MTR4 knockdown downregulates cell cycle genes and triggers cell cycle exit. Although BUB3 dysregulation has been linked to cellular senescence and age‐related pathologies in multiple model systems (Baker et al. 2006; de Magalhães et al. 2024; Kalitsis et al. 2005), whether BUB3 regulates VEC senescence and its association with MTR4 is previously unreported. Future studies will further define the precise roles of the MTR4‐BUB3 complex in spindle assembly checkpoint regulation, genome stability, and core senescence pathways including p53/p21 and p16/Rb.

We uncovered a post‐transcriptional mechanism that controls MTR4 levels during endothelial senescence, which involves SYVN1‐mediated ubiquitination at K638 and subsequent proteasomal degradation. SYVN1 is an E3 ubiquitin ligase best known for its roles in endoplasmic reticulum‐associated degradation, cell survival, differentiation, and stress responses (Zhu et al. 2025). Although previous studies have reported that SYVN1 promotes MTR4 degradation (Wang et al. 2021), our work is the first to reveal that this interaction is dysregulated in the context of cellular senescence. SYVN1 is upregulated in senescent HUVECs, and its knockdown restored MTR4 expression in aged cells, whereas SYVN1 overexpression reduced MTR4 levels in young cells. We mapped the critical ubiquitination site to K638 within the rRNA pro‐arch domain of MTR4, a residue highly conserved across vertebrates, underscoring the evolutionary relevance of this regulatory node. Importantly, our K638R rescue experiment provides direct causal evidence that blocking MTR4 degradation at this specific site is sufficient to reverse senescence. The degradation‐resistant K638R mutant rescues the senescent phenotype to the same degree as wild‐type MTR4 re‐expression. SYVN1 inhibition alleviates senescence primarily through restoring MTR4 protein levels.

We next examined whether the regulation we observed in cultured HUVECs operates in vivo. In mice, endothelial Mtr4 knockdown promoted vascular dysfunction and systemic aging phenotypes, whereas endothelium‐specific Mtr4 overexpression in aged mice alleviated these age‐related changes. Notably, endothelial Mtr4 overexpression in aged mice not only restored vascular function but also attenuated morphological deterioration in multiple organs and reduced systemic inflammation. To assess whether MTR4 function in aging is conserved across species and whether its manipulation affects lifespan, we examined the *C. elegans* homolog *mtr‐4*. *mtr‐4* mRNA levels declined with age in worms. RNAi‐mediated knockdown of *mtr‐4* shortened lifespan, accelerated age‐related functional decline, increased lipofuscin and lipid deposition, impaired stress resistance, and elevated basal ROS levels. Conversely, transgenic overexpression of *mtr‐4* extended worm lifespan. Strikingly, transgenic overexpression of *mtr‐4* doubled worm lifespan, an exceptional effect for single‐gene manipulation that is comparable to canonical longevity mutants *eat‐2(ad1116)*, a dietary restriction model, and *daf‐2(e1370)*, a core component of insulin/IGF‐1 signaling, both widely recognized as gold‐standard longevity controls. The exceptional potency of *mtr‐4* is further underscored by comparison with other single‐gene interventions, among which overexpression of *raga‐1*, *sqst‐1*, *CG11837*, *vrk‐1*, or *APL‐1* only achieves a maximum lifespan extension of 56% (Ewald et al. 2016; Kumsta et al. 2019; Park et al. 2020; Tao et al. 2024; Tuckowski et al. 2025; Zhang, Dang, and Wang 2025), whereas *mtr‐4* overexpression produced robust lifespan extension without tissue‐specific targeting. Although DAF‐16/FOXO and HLH‐30/TFEB also extend lifespan, their effects rely on dual‐pathway coordination rather than single‐gene overexpression (Ricaurte‐Perez et al. 2025). These comparisons demonstrate that *mtr‐4* overexpression elicits longevity effects comparable to classic longevity mutants and greater than most reported single‐gene interventions. Complementing these findings, the mouse data show that endothelial MTR4 influences vascular and systemic physiology, and the worm data indicate that *mtr‐4* modulates lifespan. Future studies using will be critical to determine whether MTR4 can exert such a profound lifespan‐prolonging effect in mammals as well.

Several questions remain to be addressed in future studies. First, although we have demonstrated that MTR4 interacts with BUB3 through its rRNA pro‐arch domain and stabilizes BUB3 at the protein level, the precise molecular mechanism by which MTR4 stabilizes BUB3 remains unclear. Our current data show that MTR4 maintains BUBR1 levels and limits DNA damage through BUB3, but whether MTR4‐BUB3 directly participates in BUB3's known functions in the spindle assembly checkpoint, telomere replication, or R‐loop metabolism (Li et al. 2018; Wan et al. 2015) requires further investigation. Third, whether MTR4 also contributes to endothelial dysfunction in pathological conditions such as atherosclerosis and diabetic vasculopathy remains unexplored. Furthermore, clinical samples will be required to determine whether MTR4 levels in the vascular endothelium are reduced in elderly or atherosclerotic patients.

In summary, we identify MTR4 as a conserved antiaging factor in the vascular endothelium. The senescence‐associated reduction in MTR4 is driven by SYVN1‐mediated ubiquitination at K638 and subsequent proteasomal degradation. Blocking this degradation via the K638R mutant is sufficient to reverse senescence. MTR4 attenuates endothelial senescence through Arch domain‐dependent physical interaction with BUB3, which stabilizes BUB3 and maintains BUBR1 protein levels. In *C. elegans* , *mtr‐4* overexpression alone drives striking lifespan extension that surpasses most other single‐gene interventions and is comparable to classic longevity mutants. These results were validated in HUVECs, *C. elegans* , and mice, underscoring the evolutionary conservation and strong translational potential of MTR4 as a target for age‐related vascular and multiorgan dysfunction. Our findings provide new insights into the regulatory network of VEC senescence and offer a promising strategy for endothelium‐targeted antiaging therapy.

## Author Contributions

Conceptualization: Lu Zhang; Research design: Lu Zhang; Investigation: Zhenyuan Guo, Zhiqiang Chen, Jiaqi Sun, Xiaojun Zhou, Chengzhi Ni, Yuxiang Zhang, Pengfei Qiang (performed experiments); Data analysis: Zhenyuan Guo, Lu Zhang, Zhiqiang Chen, Xiaojun Zhou; Writing – original draft: Lu Zhang and Zhenyuan Guo; Visualization: Lu Zhang and Zhenyuan Guo (figure assembly); Resources: Lu Zhang (provided facilities/materials); All authors reviewed, edited, and approved the final manuscript.

## Funding

This work was supported by Natural Science Foundation of Henan Province, 232300421030; National Natural Science Foundation of China, 32070742, 32400624; Innovative Funds Plan of Henan University of Technology, 2021ZKCJ16; Double First‐Class Discipline Construction Program of Henan University of Technology, HAUTSYL2023KC05.

## Conflicts of Interest

The authors declare no conflicts of interest.

## Supporting information


**Figure S1:** Mtr4 protein levels are downregulated in the heart, liver, spleen, lung, and kidney of aged mice (A–E) Mtr4 protein expression levels are downregulated in the heart (A), liver (B), spleen (C), lung (D), and kidney (E) of aged mice (18–20 months) compared to young (YN) mice (3–5 months), as determined by Western blotting (*n* = 4). Data are presented as mean ± SD. *p* Values were determined by unpaired two‐tailed *t*‐test with Bonferroni correction for multiple comparisons.
**Figure S2:** Comparative analysis of *Mtr4* expression between senescent and non‐senescent coronary artery endothelial cells at single‐cell resolution. Coronary artery endothelial cells from 18 to 30‐month‐old mice in the Tabula Muris Senis dataset (GSE149590) were assigned senescence scores using the hUSI system, adapted with mouse one‐to‐one orthologs via cross‐species conversion. Cells were ranked by senescence score (higher score = more senescent). The highest‐scoring 25th percentile (senescent group, *n* = 659) is denoted in blue, and the lowest‐scoring 25th percentile (non‐senescent group, *n* = 682) in red. Data are presented as mean ± SD. *p* Values were determined by unpaired two‐tailed *t*‐test with Bonferroni correction for multiple comparisons.
**Figure S3:** Transcriptomic analysis of differentially expressed genes and pathway enrichment in HUVECs upon MTR4 knockdown. (A) Volcano plot of RNA‐seq data from HUVECs after MTR4 knockdown.(B, C) Bubble plots depicting Reactome pathway enrichment analysis of significantly upregulated and downregulated genes, respectively.
**Figure S4:** MTR4 interacts with BUB3 via its Arch domain. Co‐immunoprecipitation (Co‐IP) analysis of the interaction between Flag‐BUB3 and Myc‐ΔrRNA pro‐arch (an Arch domain deletion mutant).
**Figure S5:** *MTR4* mRNA levels in different species. (A) *MTR*4 mRNA levels are upregulated in the replicative senescence (RS) model compared to proliferating (PF) HUVECs (*n* = 3). (B) *Mtr4* mRNA expression levels show no significant change in the aortas of aged mice compared to young mice (*n* = 3). (C) *mtr‐4* mRNA expression levels are decreased in aged *C. elegans* compared to young animals (*n* = 3). Data are presented as mean ± SD. *p* Values were determined by unpaired two‐tailed *t*‐test with Bonferroni correction for multiple comparisons.
**Figure S6:** *MTR4* mRNA levels in different human vascular tissues across different age groups based on the GTEx database. (A) *MTR4* mRNA levels in the aorta. (B) *MTR4* mRNA expression levels in the coronary artery. (C) *MTR4* mRNA levels in the tibial artery. (D) *MTR4* mRNA levels in the Blood vessel. No significant differences in *MTR4* mRNA levels were observed between young and aged individuals in any of the four vascular tissues examined.
**Figure S7:** Expression levels of SYVN1, P21, and P16 in young and replicative senescent HUVECs. Protein expression levels of SYVN1, P21, and P16 in replicative senescent (RS) HUVECs compared to Proliferating (PF) HUVECs, as determined by Western blotting (*n* = 3). Data are presented as mean ± SD. *p* Values were determined by unpaired two‐tailed *t*‐test with Bonferroni correction for multiple comparisons.
**Figure S8:** AAV‐mediated Mtr4 knockdown and overexpression in mice. (A) Knockdown efficiency of Mtr4 following AAV‐mediated knockdown. AAV was administered via tail vein injection in 5‐month‐old mice, and Mtr4 protein expression levels were detected by Western blotting 3 months later, with a booster injection given during this period (*n* = 10). Data are presented as mean ± SD. *p* Values were determined by one‐way ANOVA followed by Dunnett's multiple comparisons test. (B) Overexpression efficiency of Mtr4 following AAV‐mediated overexpression. AAV was administered via tail vein injection in 15‐month‐old mice, and Mtr4 protein expression levels were detected by Western blotting 3 months later, with a booster injection given during this period (*n* = 10). Data are presented as mean ± SD. *p* Values were determined by unpaired two‐tailed *t*‐test with Bonferroni correction for multiple comparisons.
**Figure S9:** AAV‐mediated Mtr4 knockdown and overexpression in mice show no significant change in body weight (A) No significant change in body weight was observed following AAV‐mediated Mtr4 knockdown in mice. (B) No significant change in body weight was observed following AAV‐mediated Mtr4 overexpression in mice.
**Figure S10:** Effects of Mtr4 knockdown and overexpression on aortic arch blood flow velocity and abdominal aortic diameter. (A, B) Representative pulse‐wave Doppler images of the aortic arch and representative B‐mode images of the abdominal aorta following Mtr4 knockdown in mice (*n* = 10). Data are presented as mean ± SD. *p* Values were determined by one‐way ANOVA followed by Dunnett's multiple comparisons test. (C, D) Representative pulse‐wave Doppler images of the aortic arch and representative B‐mode images of the abdominal aorta following Mtr4 overexpression in mice (*n* = 10). Data are presented as mean ± SD. *p* Values were determined by unpaired two‐tailed *t*‐test with Bonferroni correction for multiple comparisons.
**Figure S11:** Effects of Mtr4 knockdown and overexpression on histology of major organs in mice. (A) Representative H&E staining images of the heart, liver, spleen, lung, and kidney following Mtr4 knockdown in mice. (B) Representative H&E staining images of the heart, liver, spleen, lung, and kidney following Mtr4 overexpression in mice.(scale bar, 50 μm).
**Figure S12:** Knockdown of Mtr4 in vivo reduces Bub3 protein levels in mouse aorta. Western blotting analysis of BUB3 protein expression in aortic tissues from mice injected with AAV‐shCtr (control), AAV‐shMtr4‐1, or AAV‐shMtr4‐2 (*n* = 5). Data are presented as mean ± SD. *p* Values were determined by one‐way ANOVA followed by Dunnett's multiple comparisons test.

**Table S1:** Detailed list of materials used in this study.

**Movie S1:** Representative video showing spontaneous movement of day‐7 adult N2 wild‐type worms following control RNAi.

**Movie S2:** Representative video showing spontaneous movement of day‐7 adult N2 wild‐type worms following *mtr‐4* RNAi.

**Movie S3:** Representative video showing spontaneous movement of day‐7 adult N2 wild‐type worms.

**Movie S4:** Representative video showing spontaneous movement of day‐7 adult N2 wild‐type worms overexpressing *mtr‐4*.

## Data Availability

The RNA sequencing data from this study have been deposited in the GEO database (accession number GSE326321), and the mass spectrometry‐based interactome data of MTR4 have been deposited in the ProteomeXchange database (accession number PXD076350).
