# Supplementary material for: MTR4: A Conserved Youth Factor for Vascular Endothelium
Source: Aging Cell. 2026 Oct 16;25(10):e70774. doi: 10.1111/acel.70774 (PMC13673228; doi:10.1111/acel.70774)
Supplement: Supplementary file 1 [file ACEL-25-e70774-s005.docx]

**MTR4: A conserved youth factor for vascular endothelium**

Zhenyuan Guo^1^, Zhiqiang Chen^1^, Jiaqi Sun^1^, Xiaojun Zhou^1^, Chengzhi Ni^1^, Yuxiang Zhang^1^, Pengfei Qiang^1^, Lu Zhang^1,*^

^1^College of Bioengineering, Henan University of Technology, Lianhua Street, Zhengzhou 450001, China

* Correspondence: [zhanglu@haut.edu.cn](mailto:zhanglu@haut.edu.cn) (Lu Zhang)

Address: 100 Lianhua Street, High-tech Zone, Zhengzhou, China 450001

Tel: 0086 371 67756513

**Figure S1. Mtr4 protein levels are downregulated in the heart, liver, spleen, lung, and kidney of aged mice**

(A-E) Mtr4 protein expression levels are downregulated in the heart (A), liver (B), spleen (C), lung (D), and kidney (E) of aged mice (18-20 months) compared to young (YN) mice (3-5 months), as determined by Western blotting (n = 4). Data are presented as mean±SD. P values were determined by unpaired two-tailed *t*-test with Bonferroni correction for multiple comparisons.

**Figure S2. Comparative analysis of *Mtr4* expression between senescent and non-senescent coronary artery endothelial cells at single-cell resolution.**

Coronary artery endothelial cells from 18 to 30-month-old mice in the Tabula Muris Senis dataset (GSE149590) were assigned senescence scores using the hUSI system, adapted with mouse one-to-one orthologs *via* cross-species conversion. Cells were ranked by senescence score (higher score = more senescent). The highest-scoring 25th percentile (senescent group, n = 659) is denoted in blue, and the lowest-scoring 25th percentile (non-senescent group, n = 682) in red.Data are presented as mean±SD. P values were determined by unpaired two-tailed *t*-test with Bonferroni correction for multiple comparisons.

**Figure S3. Transcriptomic analysis of differentially expressed genes and pathway enrichment in HUVECs upon MTR4 knockdown.**

(A) Volcano plot of RNA-seq data from HUVECs after MTR4 knockdown.(B, C) Bubble plots depicting Reactome pathway enrichment analysis of significantly upregulated and downregulated genes, respectively.

**Figure S4. MTR4 interacts with BUB3 *via* its Arch domain.**

Co-immunoprecipitation (Co-IP) analysis of the interaction between Flag-BUB3 and Myc-ΔrRNA pro-arch (an Arch domain deletion mutant).

**Figure S5 . *MTR4* mRNA levels in different species**

(A) *MTR*4 mRNA levels are upregulated in the replicative senescence (RS) model compared to proliferating (PF) HUVECs (n = 3). (B) *Mtr4* mRNA expression levels show no significant change in the aortas of aged mice compared to young mice (n = 3). (C) *mtr-4* mRNA expression levels are decreased in aged *C.elegans* compared to young animals (n = 3). Data are presented as mean±SD. P values were determined by unpaired two-tailed *t*-test with Bonferroni correction for multiple comparisons.

**Figure S6. *MTR4* mRNA levels in different human vascular tissues across different age groups based on the GTEx database**

(A) *MTR4* mRNA levels in the aorta. (B) *MTR4* mRNA expression levels in the coronary artery. (C) *MTR4* mRNA levels in the tibial artery. (D) *MTR4* mRNA levels in the Blood vessel. No significant differences in *MTR4* mRNA levels were observed between young and aged individuals in any of the four vascular tissues examined.

**Figure S7. Expression levels of SYVN1, P21, and P16 in young and replicative senescent HUVECs**

Protein expression levels of SYVN1, P21, and P16 in replicative senescent (RS) HUVECs compared to Proliferating (PF) HUVECs, as determined by Western blotting (n = 3). Data are presented as mean±SD. P values were determined by unpaired two-tailed *t*-test with Bonferroni correction for multiple comparisons.

**Figure S8. AAV-mediated Mtr4 knockdown and overexpression in mice**

(A) Knockdown efficiency of Mtr4 following AAV-mediated knockdown. AAV was administered via tail vein injection in 5-month-old mice, and Mtr4 protein expression levels were detected by Western blotting three months later, with a booster injection given during this period (n = 10). Data are presented as mean±SD. P values were determined by one-way ANOVA followed by Dunnett's multiple comparisons test. (B) Overexpression efficiency of Mtr4 following AAV-mediated overexpression. AAV was administered via tail vein injection in 15-month-old mice, and Mtr4 protein expression levels were detected by Western blotting three months later, with a booster injection given during this period (n=10). Data are presented as mean±SD. P values were determined by unpaired two-tailed *t*-test with Bonferroni correction for multiple comparisons.

**Figure S9. AAV-mediated Mtr4 knockdown and overexpression in mice show no significant change in body weight**

(A) No significant change in body weight was observed following AAV-mediated Mtr4 knockdown in mice. (B)No significant change in body weight was observed following AAV-mediated Mtr4 overexpression in mice.

**Figure S10. Effects of Mtr4 knockdown and overexpression on aortic arch blood flow velocity and abdominal aortic diameter**

(A-B) Representative pulse-wave Doppler images of the aortic arch and representative B-mode images of the abdominal aorta following Mtr4 knockdown in mice (n = 10). Data are presented as mean±SD. P values were determined by one-way ANOVA followed by Dunnett's multiple comparisons test. (C-D) Representative pulse-wave Doppler images of the aortic arch and representative B-mode images of the abdominal aorta following Mtr4 overexpression in mice (n = 10). Data are presented as mean±SD. P values were determined by unpaired two-tailed *t*-test with Bonferroni correction for multiple comparisons.

**Figure S11. Effects of Mtr4 knockdown and overexpression on histology of major organs in mice**

(A) Representative H&E staining images of the heart, liver, spleen, lung, and kidney following Mtr4 knockdown in mice. (B) Representative H&E staining images of the heart, liver, spleen, lung, and kidney following Mtr4 overexpression in mice.(scale bar, 50 μm).

**Figure S12. Knockdown of Mtr4 in *vivo* reduces Bub3 protein levels in mouse aorta.**

Western blotting analysis of BUB3 protein expression in aortic tissues from mice injected with AAV-shCtr (control), AAV-shMtr4-1, or AAV-shMtr4-2 (n = 5). Data are presented as mean±SD. P values were determined by one-way ANOVA followed by Dunnett's multiple comparisons test.
