# Supplementary material for: MTR4: A Conserved Youth Factor for Vascular Endothelium
Source: Aging Cell. 2026 Oct 16;25(10):e70774. doi: 10.1111/acel.70774 (PMC13673228; doi:10.1111/acel.70774)
Supplement: Supplementary file 2 [file ACEL-25-e70774-s006.docx]

**Supplementary table 1** Detailed list of materials used in this study.

| **Antibodies (Dilution)** | **Source** | **Identifier** |
| --- | --- | --- |
| MTR4 (1:1000) | Proteintech | Cat#12719-2-AP |
| MTR4 (1:500) | ABclonal | Cat#A5576 |
| SYVN1 (1:500) | aladdin | Cat#Ab130086 |
| FEN1 (1:500) | ABclonal | Cat#A8999 |
| BUBR1 (1:1000) | zenbio | Cat#R26486 |
| BUB3 (1:1000) | zenbio | Cat#R383152 |
| BUB3 (1:10000) | Selleck | Cat#D16P3 |
| GAPDH (1:4000) | Proteintech | Cat#10494-1-AP |
| Flag-tag (1:4000) | Proteintech | Cat#20543-1-AP |
| Myc-tag (1:4000) | Proteintech | Cat#16286-1-AP |
| p16 (1:1000) | Proteintech | Cat#10883-1-AP |
| P21 (1:1000) | Proteintech | Cat#10355-1-AP |
| P53 (1:5000) | Proteintech | Cat#10442-1-AP |
| P53 (1:5000) | ABclonal | Cat#A25915 |
| ubiquitin (1:1000) | Proteintech | Cat#10201-2-AP |
| eNOS (1:5000) | Proteintech | Cat#27120-1-AP |
| Phospho-H2A.X (1:200) | Servicebio | Cat#GB111841 |
| IRDye 800CW goat anti-mouse lgG(H+L) (1:10000) | LICOR | Cat#926-32210 |
| IRDye 800CW goat anti-rabbit lgG(H+L) (1:10000) | LICOR | Cat#926-32211 |
| **Reagents** | **Source** | **Identifier** |
| Anti-Myc Magnetic Beads | Biolinkedin | Cat#L-1102A |
| Protein A+G Agarose | Beyotime | Cat#P2012 |
| Endothelial Cell Medium | ScienCell | Cat#1001 |
| DMEM High Glucose | Servicebio | Cat#G4512-500Ml |
| Opti-MEM^TM^ | Gibco | Cat#31985-070 |
| Fetal bovine serum (FBS) | Servicebio | Cat#G8002-100ML |
| PEI | Sigma-Aldrich | Cat#[919012](https://www.sigmaaldrich.cn/CN/zh/product/aldrich/919012) |
| Polybrene | Beyotime | Cat#C0351 |
| 5-fluorouracil | TargetMol | Cat#T0984 |
| DMSO | Solarbio | Cat#D8375 |
| CA-5f | TargetMol | Cat#T5181 |
| Bafilomycin A1 | MCE | Cat#HY-100558 |
| Wortmannin | MCE | Cat#HY-10197 |
| MG-132 | TargetMol | Cat#T2154 |
| Cycloheximide | MCE | Cat#HY-12320 |
| Dactinomycin D | TargetMol | Cat#TN9827 |
| TritonX-100 | Solarbio | Cat#T8200 |
| ChamQ Universal SYBR qPCR Master Mix | Vazyme | Cat#Q711-02 |
| T4 ligase | Abclonal | Cat#RM21501 |
| SanPrep Column DNA Gel Extraction Kit | Sangon Biotech | Cat#B518131 |
| EndoFree Plasmid Midi Kit | CWBIO | Cat#CW21055 |
| RaPure Total RNA Kit | Magen | Cat#R4011-02 |
| NGzol RNA Uptake Kit | HLINGENE | Cat#NG304S |
| Senescence β-Galactosidase Staining Kit | Beyotime | Cat#C0602 |
| Senescence β-Galactosidase Staining Kit | Solarbio | Cat#G1580 |
| Ready-to-Use Seamless Cloning Kit | Sangon Biotech | Cat#B632219 |
| **Cells** | **Source** | **Identifier** |
| HEK293T cells | United States National Culture Collection | Cat#CBP60439 |
| HUVECs | the Cell Bank of the Chinese Academy of Sciences | Cat#PSC-01 |
| C166 | Shanghai Zhong Qiao Xin Zhou Biotechnology Co.,Ltd. | Cat#ZQ1057 |
| DH-5α | Shanghai Weidi Biotechnology Co., Ltd. | Cat#DL1001 |
| HT115 (DE3) | Shanghai Weidi Biotechnology | Cat#EC2010 |
| **Animals** | **Source** | **Identifier** |
| Caenorhabditis elegans:N2(WT) | Professor Ding Chunbang from Sichuan Agricultural University,China | N/A |
|  |  |  |
|  |  |  |
| Caenorhabditis elegans:N2(oemtr-4) | Fujian Sunybiotech Co., Ltd. | Project No:ZLU05-06 |
| Caenorhabditis elegans：eat-2(ad465) mutant | Professor Fei Jian from Laboratory of Molecular Cell Biology, Institute of Biochemistry and Cell Biology, Model Organism Research Center, SIBS, CAS, Shanghai, China. | N/A |
| Caenorhabditis elegans：daf-2(e1370) mutant | Professor Dong Mengqiu from Beijing Key Laboratory of the Cell Biology of Animal Aging, Beijing, China. | N/A |
| Mouse: WT C57BL6/J | Laboratory Animal Center of Zhengzhou University (Henan, China) | approval No. ZZU-LAC20250219[01] |
| **plasmids** | **source** | **Identifier** |
| pHAGE-CMV-MCS-PGK-3×Flag | Our lab | N/A |
| pHAGE-CMV-MCS-PGK-3×Myc | Our lab | N/A |
| psPAX2 | Addgene | Cat#12260 |
| pMD2.G | Addgene | Cat#12259 |
| pLKO.1-TRC Cloning Vector | Addgene | Cat#10878 |
| L4440 | Addgene | Cat#1654 |
| pAAV-ICAM2-Mtr4-GFP | Wuhan Miaoling Biotechnology Co., Ltd | Project No:G61155 |
| pAAV-U6-Mtr4-shRNA1 | Wuhan Miaoling Biotechnology Co., Ltd | Project No:G62259 |
| pAAV-U6-Mtr4-shRNA2 | Wuhan Miaoling Biotechnology Co., Ltd | Project No:G62260 |
| **Plasmids primer** | **Forward primer** | **Reverse primer** |
| Myc-MTR4 | gatctggcccgggcgggatccATGGCGGACGCATTCGGA | cttgtaatcctcgaggtcgacCTACAAGTAGAGGCTGGCAGCA |
| Flag-SYVN1 | CGCACGCGTTGCCACCATGTTCCGCACGGCAGTG | CGCGTCGACGTGGGCAACAGGAGACTCCA |
| Myc-MTR4-DEAD | gatctggcccgggcgggatccATGGCGGACGCATTCGGA | cttgtaatcctcgaggtcgacCTAAAACTGTCGGGCATTTGG |
| Myc-MTR4-HelicaseC | gatctggcccgggcgggatccGCTGAATGGATTTGCCATTTACA | cttgtaatcctcgaggtcgacCTATCCTCTCCTTCCAGCACGA |
| Myc-MTR4-rRNA pro-arch | gatctggcccgggcgggatccATGGATGATAGAGGAATTGTAATTCTTATG | cttgtaatcctcgaggtcgacCTATTTCTTCAGTTCTCGCTTTGC |
| Myc-MTR4-DSHCT | gatctggcccgggcgggatccGCAAGAACAGTCCTACAAATGGATG | cttgtaatcctcgaggtcgacCTACAAGTAGAGGCTGGCAGCA |
| Myc-MTR4-ΔDEAD | gatctggcccgggcgggatccGCTGAATGGATTTGCCATTTACA | cttgtaatcctcgaggtcgacCTACAAGTAGAGGCTGGCAGCA |
| Myc-MTR4-ΔHelicaseC | ccgacagtttatggatgatagaggaattgta | ctatcatccataaactgtcgggcatttggaatag |
| Myc-MTR4-ΔrRNA pro-arch | aaggagaggagcaagaacagtcctacaaatggat | actgttcttgctcctctccttccagcacgac |
| Myc-MTR4-ΔDSHCT | gatctggcccgggcgggatccATGGCGGACGCATTCGGA | cttgtaatcctcgaggtcgacCTATTTCTTCAGTTCTCGCTTTGC |
| Myc-MTR4-MTR4-K638R | cagcttgccaaattgggtaGagaaattgaagaatatattc | gaatatattcttcaatttctCtacccaatttggcaagctg |
| Flag-MYC | CGCacgcgtctggatttttttcgggtagtggaaa | CGCgtcgaccgcacaagagttccgtagct |
| Flag-CDK1 | CGCacgcgtGCCACCatggaagattataccaaaatagagaaa | CGCgtcgaccatcttcttaatctgattgtccaaa |
| Flag-AIFM1 | CGCacgcgtGCCACCatgttccggtgtggaggcct | CGCgtcgacgtcttcatgaatgttgaatagttt |
| Flag-BUB3 | CGCacgcgtGCCACCatgaccggttctaacgagttcaag | CGCgtcgacggtggacttgggttttgtttctg |
| Flag-FEN1 | CGCacgcgtGCCACCatgggaattcaaggcctggc | CGCgtcgacttttccccttttaaacttccctgctg |
| **pAAV-ICAM2-Mtr4-GFP** | **Forward primer** | **Reverse primer** |
| ICAM2-Mtr4 | ccaggcagcccttggggatccATGGCGGATGCTTTCGGG | ccttgctcaccatggggatccTAAGTAGAGGCTGGCAGCAAATAC |
| **pLKO.1 primer** | **gRNA** |  |
| MTR4 shRNA | AGCTAAAGGAATGTGAGATTT |  |
| MTR4 shRNA#1 | CCCTTATTAGACCCTATTGAT |  |
| MTR4 shRNA#2 | GCTCCGCTGATCCTCTAAATA |  |
| SYVN1 shRNA#1 | GCTCACGCCTACTACCTCAAA |  |
| SYVN1 shRNA#2 | GACCGTGTGGACTTTATGGAA |  |
| **pAAV-U6-Mtr4-shRNA-ZsGreen1** | **gRNA** |  |
| Mtr4 shRNA#1 | AGGATTGATAAAGGCTTTATT |  |
| Mtr4 shRNA#2 | CCATCAAATGTGTTCAAGATT |  |
| C. elegans L4440 | **Forward primer** | **Reverse primer** |
| *mtr-4*#1 | gtcgacggtatcgataagcttTGCACAATGATGTCGGAGTTGG | caggaattcgatatcaagcttTTGGACGATAGTCTGTGTAAACCAC |
| *mtr-4*#2 | gtcgacggtatcgataagcttGATATATCACGTCAGGAGAATACATTCA | caggaattcgatatcaagcttGCATTTGTCCATCTGGCTTATCA |
| **Gene** | **Forward primer** | **Reverse primer** |
| MTR4 | CCACGAGTTGGAAAAGCTGC | AATGGCATACTCGGCGCATA |
| FEN1 | TCACTAAGCGGCTGCTAAG | CAGTCCATGTCCTCGGTAGC |
| BUB3 | CACGAGCATCGCATCACTTG | GGATGATTAGGTGGACTTGGGT |
| IL-1 | AACCTCTTCGAGGCACAAGG | GTCCTGGAAGGAGCACTTCAT |
| IL-6 | TTCTCCACAAGCGCCTTC | CCGTCGAGGATGTACCGAAT |
| TNF-α | GTAGCCCATGTTGTAGCAAACC | GGACCTGGGAGTAGATGAGGT |
| MMP3 | ACAAAGGATACAACAGGGACCA | ATCTTGAGACAGGCGGAACC |
| CCL2 | GAGAGGCTGAGACTAACCCAG | GGGCATTGATTGCATCTGGC |
| TGF-β | CTGGCGATACCTCAGCAACC | GCCGGTAGTGAACCCGTTG |
| GAPDH | TCGGAGTCAACGGATTTGGT | TTCCCGTCTCAGCCTTGAC |
| mtr-4(C) | CGAGATAAAGAGCGCGGAGT | TTCGCGGAATTCACCCTTCA |
| *actin-1*(C) | CAAGAGAGGTATCCTTACCCTCAAG | GTCCGGAAGCGTAGAGGGAG |
| Mtr4(m) | TGCTTTCGGGGATGAGCTTT | GTATCCAGTCGTTTCCCGGC |
| Gapdh(m) | AAGAGGGATGCTGCCCTTAC | TACGGCCAAATCCGTTCACA |
| **Softwares** | **Source** |  |
| Graphpad Prism for statistical analysis | GraphPad |  |
| ImageJ | NIH |  |
| Image Studio | Alias |  |
| Snapgene Viewer | GSL Biotech LLC |  |
| LI-COR Odyssey system | LI-COR |  |
| Zen | ZEISS |  |
| ImageView | ToupTek |  |
| CaseViewer | 3DHISTECH |  |
