# Supplementary material for: MTR4: A Conserved Youth Factor for Vascular Endothelium
Source: Aging Cell. 2026 Oct 16;25(10):e70774. doi: 10.1111/acel.70774 (PMC13673228; doi:10.1111/acel.70774)
Supplement: Supplementary file 5 [file ACEL-25-e70774-s003.zip › Movie S1.docx]

**Movie S1:** Representative video showing spontaneous movement of day-7 adult N2 wild-type worms following control RNAi.
